# Transcriptome datasets of wheat plant cultures treated with seed priming fertilizer

**DOI:** 10.1016/j.dib.2025.112050

**Published:** 2025-09-10

**Authors:** Kincső Decsi, Mostafa Ahmed, Roquia Rizk, Donia Abdul-Hamid, Zoltán Tóth

**Affiliations:** a Institute of Agronomy, Georgikon Campus, Hungarian University of Agriculture and Life Sciences, 8360 Keszthely, 7 Festetics street, Hungary; b Festetics Doctoral School, Institute of Agronomy, Georgikon Campus, Hungarian University of Agriculture and Life Sciences, 8360 Keszthely, 16 Deák Ferenc Street, Hungary; c Department of Agricultural Biochemistry, Faculty of Agriculture, Cairo University, Giza 12613, University Street, Egypt; d Heavy Metals Department, Central Laboratory for The Analysis of Pesticides and Heavy Metals in Food (QCAP), Dokki, Cairo 12311, 77 Nadi El-said Street, Egypt

**Keywords:** Seed priming fertilizer, Wheat, Illumina NGS, RNA-seq, Priming

## Abstract

Extreme weather conditions cause stress in agriculture. To prevent these stress effects, techniques are now available that focus on prevention. One of these solutions could be the use of priming agents, which prepare plants for stress effects, as their application can trigger plant defense responses. Wheat plants were treated with seed priming fertilizer in advance and then exposed them to drought stress during the growing season. Deep RNA sequencing was performed using the NGS technique, which sequences - after processing and evaluation - are suitable for examining the stimulatory effects of seed priming and for monitoring the long-term effects of priming during stress effects. Four forward and reverse SRA data sets were deposited in NCBI. In addition, a file - containing the *de novo* transcriptome assembled from the cleaned and filtered raw reads (Transcriptome Shotgun Assembly - TSA) and the count table from the RNA-sequencing read quantification (Count table) - were deposited in the Mendeley database. Future results from the databases will be suitable for the immediate (priming) and long-term (under stress conditions) study of the effects of treatments and will allow the identification and monitoring of stimulated plant biochemical processes - based on gene expression changes.

Specifications Table

**Table:** 

Subject	Plant Science: Plant Physiology
Specific subject area	NGS RNA sequencing, data cleaning and preprocessing were performed on leaf samples of wheat plants from a greenhouse pot experiment, which were pre-treated with seed priming fertilizer and then subjected to drought stress.
Type of data	Filtered, preprocessed datasets Tables Analyzed data
Data collection	For the experiments, seeds of wheat plants of the Kinachi-97 variety were treated (primed) with a simple, commonly used macro- and micronutrient seed fertilizer, the active ingredients of which were as follows: P_2_O_5_: 250 g/kg, K_2_O: 170 g/kg, B: 2.5 g/kg, Cu: 1.75 g/kg, Fe: 35 g/kg, Mn: 30 g/kg, Mo: 0.25 g/kg, Zn: 32.5 g/kg). For bioinformatics studies, 30–50 mg of leaf samples were taken from each well-developed, healthy wheat plant in 4-4 replicates per treatment. The collected samples were pre-evaluated for the NGS (Next Generation Sequencing) technique (only leaf tissue samples containing total RNA with a RIN value of ≥ 7 could be used for further steps). mRNA was purified from the good quality samples (with paramagnetic NEXTFLEX® Poly(A) Beads 2.0), then after fragmentation, strand-specific NGS library preparation was performed using the NEXTFLEX® Rapid Directional RNA-Seq 2.0 kit. The resulting pooled libraries were sequenced on the Illumina NovaSeq 6000 next-generation genome sequencing platform using deep sequencing technique (2 × 150 bp PE reads; average paired-end read count 50 million/sample) [1].
Data source location	Data were collected and stored in Keszthely, Hungary, in the greenhouse of the Institute of Agronomy of the Hungarian University of Agricultural and Life Sciences, Georgikon Campus.
Data accessibility	The Bioproject and the SRA-s (RNA-seq reads) are available in National Center for Biotechnology Information (NCBI) database under the accessions: Repository name: Wheat treated by ZnO nanoparticles or by seed priming conditioner Data identification number: PRJNA1142041 Direct URL to data: https://www.ncbi.nlm.nih.gov/bioproject/PRJNA1142041 Repository name: Wheat_1_R1 and Wheat_1_R2 (control plants) Data identification number: SRR30042189 Direct URL to data: https://www.ncbi.nlm.nih.gov/sra/?term=SRR30042189 Repository name: Wheat_5_R1 and Wheat_5_R2 ( drought stressed plants) Data identification number: SRR30042187 Direct URL to data: https://www.ncbi.nlm.nih.gov/sra/?term=SRR30042187 Repository name: Wheat_17_R1 and Wheat_17_R2 (seed priming treated plants) Data identification number: SRR30042184 Direct URL to data: https://www.ncbi.nlm.nih.gov/sra/?term=SRR30042184 Repository name: Wheat_21_R1 and Wheat_21_R2 (seed priming and drought stressed plants) Data identification number: SRR30042188 Direct URL to data: https://www.ncbi.nlm.nih.gov/sra/?term=SRR30042188 TSA (Transcriptome Shotgun Assembly) and count table from the RNA-seq read quantification are deposited in the Mendeley database and named in this article as Supplementary_files. Repository name: Decsi et al., 2025_DIB_article Data identification number: doi:10.17632/y6zf546s4t.1 Direct URL to data: https://data.mendeley.com/datasets/y6zf546s4t/1
Related research article	Rizk, R.; Ahmed, M.; Abdul-Hamid, D.; Zedan, M.; Tóth, Z.; Decsi, K. Resulting Key Physiological Changes in *Triticum aestivum* L. Plants Under Drought Conditions After Priming the Seeds with Conventional Fertilizer and Greenly Synthesized Zinc Oxide Nanoparticles from Corn Wastes. *Agronomy***2025**, *15*, 211. https://doi.org/10.3390/agronomy15010211 Decsi, K.; Ahmed, M.; Abdul-Hamid, D.; Rizk, R.; Tóth, Z. Verification of Seed-Priming-Induced Stress Memory by Genome-Wide Transcriptomic Analysis in Wheat (*Triticum aestivum* L.). *Agronomy***2025**, *15*, 1365. https://doi.org/10.3390/agronomy15061365

## 1 Value of the Data

• Nowadays, the increase in the number of abiotic stress situations caused by climate change is a major problem worldwide. These factors can significantly reduce the efficiency of agricultural production. So far, efforts have mostly been aimed at remediation, but we have not focused enough on prevention.
• We have known for a long time that plants are able to respond to environmental changes with coordinated defense responses using signal transduction pathways. Jones and Dangl (2006) also described the functioning of the plant immune system in their study [2].Since plants have an immune system, the possibility has arisen that this immune system can be stimulated by substances consisting of certain biologically active components. This process is called priming and the reactions triggered in plants are called priming reactions [3].
• Seed priming fertilizers are popular among priming compounds, which exert their positive effects starting from early phenophases. The seed priming fertilizer we tested is a commonly used seed fertilizer, and we investigated its possible immunostimulatory effects in our treatments.
• Priming compounds exert a weak stimulatory effect (eustress) on the plant immune system, thereby inducing the plant to initiate defense responses. Such responses are always associated with gene-level transcriptomic changes. The investigation of the priming effects of seed priming preparations and the subsequent inducibility of priming effects - i.e. the exploration of stress memory processes - are still under-researched areas.
• Our studies provide insight into the understanding of gene-level inductive defense responses and the possibilities of their re-induction, thereby allowing researchers to come closer to understanding the functioning of the plant immune system.
• Our datasets provide support for genome-wide analyses and may even be suitable for further investigation of individual biochemical pathways or individual transcription factors and genes involved in stress responses.

## 2 Background

The use of seed priming preparations is widespread today, yet few people know that their effect is mainly stimulatory, acting as a mild stressor. Researchers have so far paid little attention to studying the effects of such preparations at the gene level.

Our goal was to explore the effects of treatments during priming and longer-term stress, and to study and gain a deeper understanding of transcriptional processes.

## 3 Data Description

In one approach, we treated wheat seeds with seed priming fertilizer before germination and examined the priming effects at the gene level.

In the other approach, we subjected the previously seed priming plants to drought stress at a later phenological phase and examined at the gene level whether their stress memory could be recalled.

Plant material used: seeds of the Kinachi 97 wheat variety.

The active ingredients of the seed priming fetilizer used by us were as follows:

P_2_O_5_: 250 g/kg, K_2_O: 170 g/kg, B: 2.5 g/kg, Cu: 1.75 g/kg, Fe: 35 g/kg, Mn: 30 g/kg, Mo: 0.25 g/kg, Zn: 32.5 g/kg) (Dr Green seed priming, Chrzanów, Poland). The seeds were soaked in a solution of 10 g/250 mL of seed priming agent for 24 hours at 25 °C in the dark with continuous moderate agitation. The treatments were as follows:

— Control plants (Wheat_control_1.fastq and Wheat control_2.fastq)
— Drought-stressed plants (Wheat_control_under_drought_stress_1.fastq and Wheat_control_under_drought_stress_2.fastq)
— Seed priming treated plants (Wheat_seed_priming_1.fastq and Wheat_seed_priming_2.fastq)
— Seed priming and drought stressed plants (Wheat_seed_priming_under_drought_stress_1.fastq and Wheat_seed_priming_under_drought_stress_2.fastq)

The plants were planted in pots placed in the university greenhouse, in a homogeneous 1:1 mixture of Ramann-type brown forest soil typical of the area and peat. The drought stress was ensured by watering with half the water doses compared to the control. After RNA isolation and library preparation from leaf samples collected from control and treated plant stands, SRA data from raw reads were deposited in the NCBI database.

**Fig. 1:**
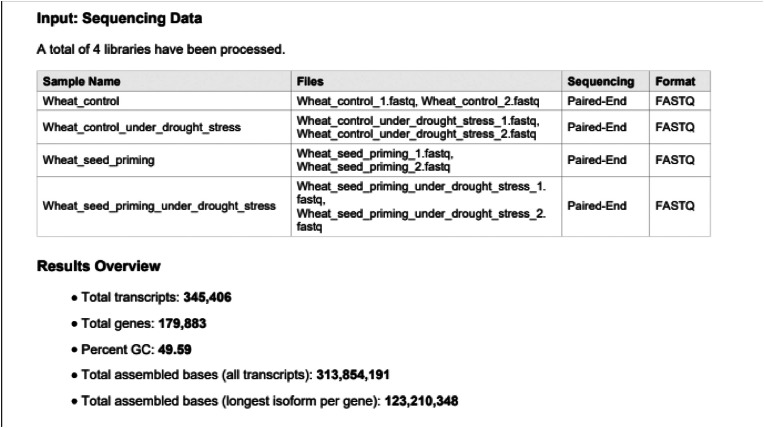
Statistical overview of *de novo* transcriptome assembly. (The figure shows the characteristic data of the de novo transcriptome assembled by the software, such as the four libraries required for assembly, and the quantitative characteristics of the transcriptome obtained from them).

After pre-screening the samples, a *de novo* transcriptome was generated using 313.854.191 short reads. The short reads were derived from the bulked biological samples sequenced of four replicates per treatment (Fig. 1). The *de novo* transcriptome reconstruction resulted in 345.406 transcripts, from which 179.883 identified genes were determined. The average length of the genes was 815 bp. The super transcriptome (TSA) is available in the Decsi et al. 2025_DIB_article_supplementary file1.

To determine the individual expression levels of genes that will be identified later, we first generate a count tablet derived from the *de novo* transcriptome. The count table generated as a result of RNA sequencing quantification from TSA (Transcriptome Shotgun Assembly) can be found in Decsi et al. 2025_DIB_article_supplementary file_2.

**Fig. 2:**
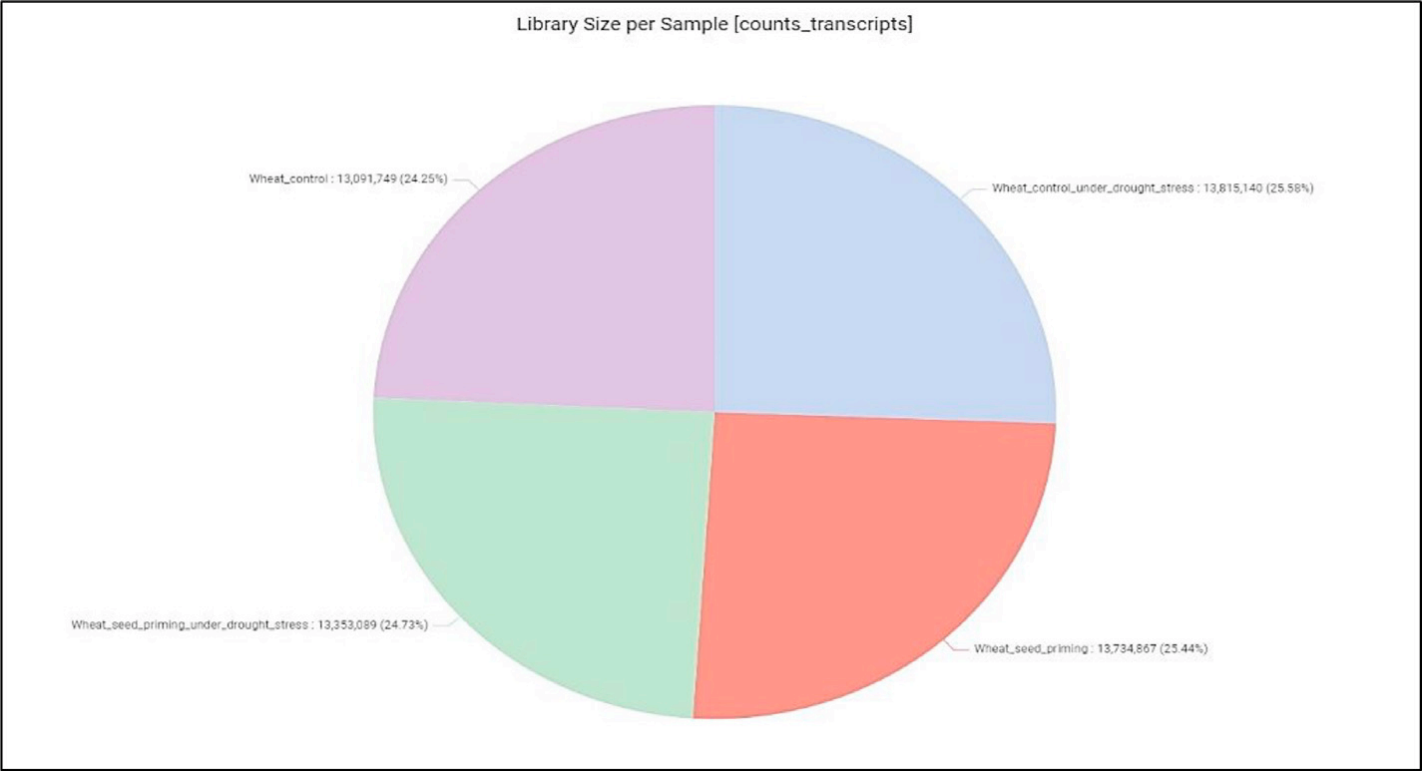
Estimated size of RNA libraries obtained from each treatment based on count table. (Captions indicate raw read libraries from each treatment).

The count table can then be used to calculate the individual gene expression levels of contigs in the de novo transcriptome. The amount of contigs (genes after identification) activated by each treatment is illustrated by the library sizes per treatment (Fig. 2).

Further analyses will allow us to identify genes activated and repressed by each treatment. After functional annotation of these genes, their participation and role in each biochemical pathway can be determined.

## 4 Experimental Design, Materials and Methods

To investigate the priming effect, 30-50 mg leaf samples were taken from healthy, young plants as soon as a plant of appropriate size was available. The soil moisture content and water holding capacity of the experimental pots were measured gravimetrically in the pots. The control and seed-prepared plants were then irrigated with the same amount of water during the growing season. Drought stress was induced by applying a half dose reduced water volume. Drought stress was applicated to mature, but not yet flowering plants, and on the 8th day after water deprivation, 30-50 mg leaf samples were also taken from the plants, 4-4 replicates per treatment. The samples were stored in 1 mL RNALater (Invitrogen by Thermo Fisher Scientific Inc., Waltham, MA, USA) solution at -20 °C until further use.

RNA was isolated from the collected leaf samples using paramagnetic NEXTFLEX® Poly(A) Beads 2.0 and then subjected to quality control. Only leaf tissue samples containing total RNA with a RIN value of ≥ 7 were used for further steps. After fragmentation, strand-specific NGS library construction was performed using the NEXTFLEX® Rapid Directional RNA-Seq 2.0 kit.

The resulting pooled libraries were sequenced using the Illumina NovaSeq 6000 next-generation genome sequencing platform using deep sequencing (2 × 150 bp PE reads; average paired-end read count 50 million/sample) [1]. The authors performed quality control on the raw reads, removed low-quality regions, and filtered the reads using FastQC (https://timkahlke.github.io/LongRead_tutorials/QC_F.html (accessed May 23, 2025)) and Trimmomatic (http://www.usadellab.org/cms/index.php?page=trimmomatic (accessed May 23, 2025)) software [4].

The quality-controlled reads were then concatenated into a *de novo* transcriptome. This was done using the available Trinity software (http://TrinityRNASeq.sourceforge.net (accessed May 23, 2025)) [5]. The Trinity sequence assembly software can assemble short nucleotide sequences into longer contigs and is unique in that it does not require a reference genome.

Trinity is a complex system consisting of three software packages that partition raw read data into unique de Bruijn graphs. Each graph represents a transcript of a given contig or gene, which is processed individually by the software. In the *de novo* assembly step, the deep sequenced raw reads are assembled non strand-specific by Bowtie2. Trinity normalizes the input reads. The reads belonging to the assembled transcript are recorded and counted by the software.

Once the *de novo* transcriptome is complete, Trinity groups the transcripts into clusters based on shared sequence content. Such a transcript cluster can be considered a - as yet unknown - “gene”. The transcript-level quantification step is used to estimate gene and isoform expression levels from RNA-sequencing data. This requires a pre-assembled *de novo* transcriptome and short raw read data.

To estimate individual expression levels per transcript, the raw reads are aligned to the *de novo* transcript. The application is based on RSEM, a software package that quantifies expression from transcriptome data. This program handles both the alignment of reads to reference transcript sequences and the calculation of relative abundances. RSEM uses the Bowtie2 aligner to align the raw reads into *de novo* transcriptome.

The individual expression levels of *de novo* transcriptome contigs must be estimated to perform differential expression analysis [6]. The software records a summary of the results in a count table output file.

## Limitations

None.

## Ethics Statement

All authors of the above scientific data reporting publication acknowledge and confirm that the authors have read and adhere to the ethical requirements for publication in Data in Brief and confirm that the current work does not involve human subjects, animal testing or data collected from social media platforms*.* We did not utilize chemicals, adjuvants, or industrial auxiliaries derived from animals. Furthermore, we do not utilize food, substances, adjuvants, or industrial auxiliaries that have been tried on animals. We did not utilize food, ingredients, adjuvants, or manufacturing auxiliaries derived from Genetically Modified Organisms (GMOs) released into the environment.

## CRediT Author Statement

**Kincső Decsi:** Writing – original draft, Conceptualization, Visualization, Validation, Supervision. **Mostafa Ahmed:** Writing—review and editing, Validation, Visualization. **Roquia Rizk:** Investigation, Validation. **Donia Abdul-Hamid:** Investigation, Validation. **Zoltán Tóth:** Supervision, Financialization.

## Data Availability

Mendeley DataDecsi et al., 2025_DIB_article (Original data)
SRR30042188Wheat_21_R1 and Wheat_21_R2 (seed priming under drought stress) (Original data)
SRR30042184Wheat_17_R1 and Wheat_17_R2 (seed priming) (Original data)
SRR30042187Wheat_5_R1 and Wheat_5_R2 (control under drought stress) (Original data)
SRR30042189Wheat_1_R1 and Wheat_1_R2 (control) (Original data)
PRJNA1142041Wheat treated by ZnO nanoparticles or by seed priming conditione (Original data)
